# Assessment of the transcriptomic consequences and MAU2 protein levels in edited induced pluripotent stem cells with *NIPBL* pathogenic variants

**DOI:** 10.1016/j.gendis.2024.101386

**Published:** 2024-08-06

**Authors:** Kévin Cassinari, Anne Rovelet-Lecrux, Céline Derambure, Myriam Vezain, Sophie Coutant, Anne-Claire Richard, Nathalie Drouot, Juliette Coursimault, Gabriella Vera, Alice Goldenberg, Pascale Saugier-Veber, Camille Charbonnier, Gaël Nicolas

**Affiliations:** a Univ Rouen Normandie, Normandie Univ, Inserm U1245 and CHU Rouen, Department of Genetics and Reference Center for Developmental Disorders, Rouen F-76000, France; b Univ Rouen Normandie, Normandie Univ, Inserm U1245 and CHU Rouen, Department of Biostatistics, F-76000 Rouen, France

Cornelia de Lange Syndrome (CdLS) is an intellectual disability syndrome characterized by distinctive clinical features including growth retardation, limb malformation, and a characteristic facial dysmorphism.^1^ Six genes, including *NIPBL* and *MAU2*, are associated with CdLS, all encoding components or partners of the cohesin protein complex. Cohesins play a central role in gene expression regulation by organizing chromatin and modulating transcription.^2^ CdLS is classified as a transcriptomopathy due to dysregulated transcription resulting from pathogenic variants in cohesin-related genes. *NIPBL* mutations are the most common cause of CdLS, impairing cohesin loading onto DNA. Previous transcriptomic studies have identified deregulated genes in CdLS (*e*.*g*., Liu et al^3^ or Mills et al^4^), but none have comprehensively assessed the impact of various *NIPBL* variants, including missense mutations, on isogenic induced pluripotent stem cells (iPSCs). In this study, we investigated mRNA and protein levels of NIPBL and MAU2, along with transcriptomic consequences of multiple *NIPBL* pathogenic variants, in isogenic iPSCs. Our findings confirm the role of *NIPBL* and *MAU2* in CdLS pathogenesis and highlight deregulated genes contributing to the syndrome's phenotype.

We selected five *NIPBL* pathogenic variants carried by patients with a typical CdLS phenotype based on the national recruitment of our molecular genetics lab in Rouen, France, namely, c.133C > T p.(Arg45^∗^) in exon 3, c.2500C > T p.(Arg834^∗^) in exon 10, c.4396dup p.(Ser1466Lysfs^∗^13) in exon 20, c.6470A > G, p.(Asp2157Gly) in exon 37, and (c.6892C > T (p.Arg2298Cys) in exon s40 (Fig. S1). We successfully introduced all five in induced pluripotent stem cells (iPSCs) by CRISPR/Cas9 editing, generating a total of 15 edited lines with either of these five different variants (see supplementary methods). For further analyses, we also kept several iPSC lines with frameshift short insertions or deletions (indels) introduced during genome editing because of aberrant DNA repair at the targeted positions. The protein-truncating variants (PTVs) in E3 were classified as early PTVs because they occur in 5′ of a suspected alternative translation initiation site. Interestingly, we could not get any clone carrying a homozygous truncating variant in *NIPBL*, suggesting that a full knockout of this gene is not compatible with iPSC survival and mitosis. According to the RNA sequencing count of linearized *NIPBL* reads (expressed in TPM), we observed a notable decrease in *NIPBL* mRNA levels in iPSC lines carrying PTVs compared to wild-type (WT), after exclusion of the early PTV, showing a 41.6% decrease (*P* < 0.001) (Fig. S2A). Interestingly, a non-significant increase of 22.4% (*P* = 0.14) in mRNA levels was detected in the iPSC line carrying the early PTV as compared to controls, suggesting that these transcripts are not degraded by nonsense-mediated decay (NMD), as previously reported.^5^ As expected, no significant difference in NIPBL mRNA levels was observed for both heterozygous (−10.0%, *P* = 0.41) and homozygous (−8.6%, *P* = 0.43) missense variants. These findings were confirmed for both PTV and early PTV variants through an independent two-step relative RT-ddPCR assay. Furthemore, the same pattern of mRNA level variation was observed, including a significant decrease in iPSC lines carrying PTV variants (−61%, *P* < 0.05), and a surge in mRNA levels for lines with early PTV variants (+39%, *P* = 0.15). Concerning *MAU2*, according to RNA sequencing data, there was no significant variation between iPSC lines carrying PTVs (after exclusion of the early PTV) and WT iPSC lines, with an observed change of only +0.01% (NS) (Fig. S2B). No significant variation in *MAU2* mRNA levels was detected in iPSC lines carrying either heterozygous or homozygous missense *NIPBL* variants. Interestingly, a non-significant increase in MAU2 mRNA levels was observed in cells carrying an early PTV. We then assessed the NIPBL and MAU2 protein levels for each clone with western blotting (Fig. 1A). As expected, NIPBL protein levels were significantly decreased in cell line carrying PTVs compared with WT (−35,8%, *P* = 0.0002), except for the early PTVs (Fig. S3). Missense variants, both homozygous and heterozygous, led to a slight but non-significant decrease in NIPBL levels. Strikingly, all iPSC mutant cell lines presented a significant decrease of MAU2 protein levels as compared with controls, including PTVs (−58,4%, *P* < 0.0001), early PTVs (−49,3%, *P* = 0.0364), heterozygous missense variants (−40.3%, *P* = 0.0005), and the homozygous missense variant (−64,8%, *P* < 0.0001). These results confirm previous findings for early PTVs and PTVs and extend those to missense variants (Fig. 1A, B). This highlights the impact of *NIPBL* alteration on MAU2 protein stability.

**Figure 1:**
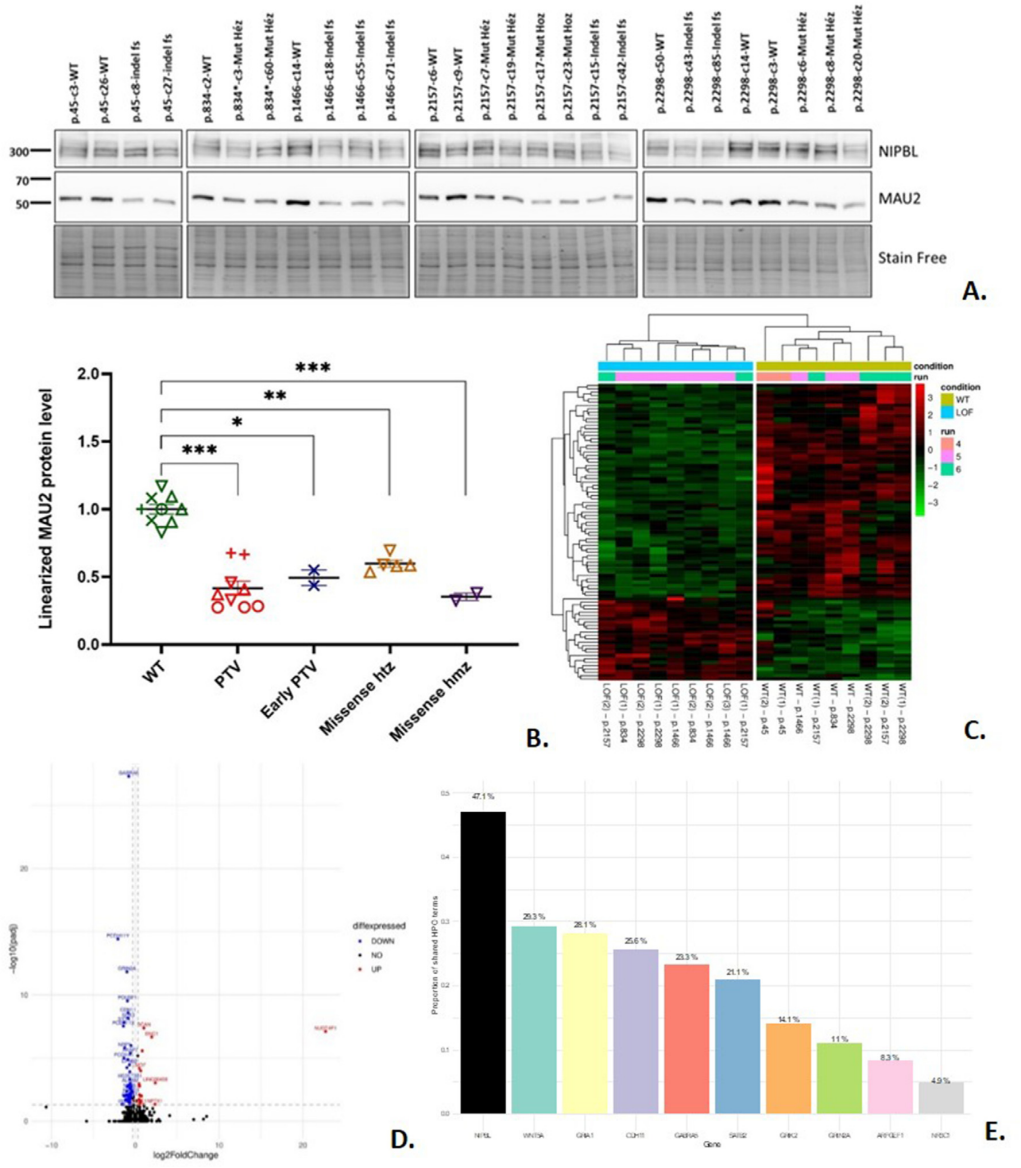
Protein and transcriptomic assessment of induced pluripotent stem cell (iPSC) line. **(A, B)** Protein analyses. (A) For each clone, the expression of NIPBL (upper panel) and MAU2 (middle panel) was assessed by western blotting. The staining was reported to the stain-free signal (lower panel), corresponding to the total amount of protein loaded on the gel. (B) Protein levels for MAU2 reported to the stain-free signal. The mean protein level of the wild-type (WT) lines was arbitrarily set at 1. For each graph, × represents variants in exon 3 (p.45), + represents variants in exon 10 (p.834), ○ represents variants in exon 20 (p.1466), ▽ represents variants in exon 37 (p.2157), and △ represents variant in exon 40 (p.2298). Green symbols in the WT bar represent the WT control of each variant. Horizontal bars represent average and standard deviation. ∗*P* < 0.05, ∗∗*P* < 0.005, ∗∗∗*P* < 0.001; NS, non-significant. **(C**–**E)** Transcriptomic analyses. (C) The heatmap presents an overview of the transcriptomic signature obtained by comparing iPSCs carrying protein-truncating variants (PTVs, with the exclusion of PTVs in exon 3, designated as PTV on the below the heatmap) to WT iPSCs. (D) The volcano plot showcasing down-regulated genes (in blue) and up-regulated genes (in red) with –log_10_(false discovery rate) on the *y*-axis. Dashed lines indicate the 1.25 fold-change cut-off and 5% false discovery rate cut-off used to declare significance. (E) Proportion of overlap between Human Phenotype Ontology (HPO) terms. For NIPBL, the ratio of the number of HPO terms shared with at least one of the nine genes with respect to the total number of NIPBL HPO terms. For each remaining gene, the ratio of the number of HPO terms shared with NIPBL with respect to their number of HPO terms.

We then assessed the consequences of *NIPBL* pathogenic variants at the transcriptome level. From RNA sequencing data, we found that 60 genes were significantly differentially expressed between PTV-iPSCs and WT controls, with a fold change >1.25 or <0.8 and at a false discovery rate below 5% (Fig. 1C, D). Of them, 46 genes were down-regulated, after the exclusion of *NIPBL* itself, and 18 of them (39%) are haploinsufficient (gnomad pLI >0.9), meaning that a single copy of these genes is not expected to lead to a normal phenotype. Here, fold changes of these 18 genes ranged from 0.64 to 0.43, which is in the same range as what is expected in case of a single copy of a given gene ( × 0.5 on average). Strikingly, 13 of the 46 down-regulated genes are associated with an OMIM Morbid phenotype and nine are both haploinsufficient and associated with an OMIM Morbid phenotype. These nine genes are also referenced in the Human Phenotype Ontology. We then looked at the list of nine genes in other mutant conditions in iPSCs. All of them were also significantly deregulated in the early-PTVs lines as well as in heterozygous and homozygous missense mutant lines, all in the same direction. Coming back to the nine genes down-regulated and considered haploinsufficient and OMIM-morbid, we compared Human Phenotype Ontology features associated with these genes to that of NIPBL-CdLS. Strikingly, the main phenotypic features of NIPBL-CdLS overlapped with that of the deregulated genes, suggesting that these genes may have a role in CdLS pathophysiology (Fig. 1E; Fig. S4). The same analysis was carried out for up-regulated genes by checking their triplosensitivity score, but none were triplosensitive (pTriplo >0.9), and only two were associated with OMIM-morbid phenotypes. Finally, we generated RNA sequencing data from the fresh blood of eight CdLS patients carrying one of the pathogenic *NIPBL* variants selected for the iPSC edition. Unfortunately, only 22 of the 60 deregulated genes in PTV-mutant iPSCs were expressed in the blood (TPM cutoff set at 1), without the same pattern, and none of the haploinsufficient plus OMIM-morbid genes belonged to this list.

In this work, we employed CRISPR/Cas9 technology to introduce various variants of the NIPBL gene into iPSCs, creating heterozygous or homozygous edited cell lines. We chose iPSCs because they provide a versatile model for examining the effects of these variants during early development. Characterization at both transcriptomic and protein levels revealed decreased levels of the MAU2 protein in all lines carrying deleterious NIPBL variations. Transcriptomic analysis performed by RNA sequencing unveiled a distinct set of up-regulated or down-regulated genes, some of which likely contribute to CdLS phenotype. While few genomic alterations of MAU2 have been described in CdLS patients, findings suggest that MAU2 loss-of-function variants could play a causative role in CdLS. The study expanded on previous research by demonstrating that various deleterious NIPBL variants, including missense mutations, were associated with decreased MAU2 protein levels. Notably, even missense variants located outside the known interaction domain with MAU2 led to reduced MAU2 levels. In this study, we utilized isogenic iPSC models to establish CdLS transcriptomic signatures, recognizing limitations regarding tissue specificity. Despite challenges, the approach allowed for the identification of down-regulated haploinsufficient genes overlapping with CdLS phenotypes, such as intellectual disability and developmental abnormalities. These findings provide valuable insights into CdLS pathogenesis and suggest potential biomarkers and therapeutic targets for further investigation.

## Ethics declaration

This study was approved by the Institutional Review Board of the Rouen University Hospital (CERDE notification E2023-65).

## Author contributions

**Kévin Cassinari:** Conceptualization, Data curation, Formal analysis, Funding acquisition, Investigation, Methodology, Project administration, Resources, Validation, Writing – original draft, Writing – review & editing. **Anne Rovelet-Lecrux:** Conceptualization, Data curation, Formal analysis, Investigation, Resources, Writing – review & editing. **Céline Derambure:** Conceptualization, Data curation, Formal analysis, Software. **Myriam Vezain:** Software. **Sophie Coutant:** Software. **Anne-Claire Richard:** Formal analysis. **Nathalie Drouot:** Formal analysis. **Juliette Coursimault:** Investigation. **Gabriella Vera:** Investigation. **Alice Goldenberg:** Investigation. **Pascale Saugier-Veber:** Conceptualization, Project administration, Supervision. **Camille Charbonnier:** Conceptualization, Methodology, Software, Validation, Writing – review & editing. **Gaël Nicolas:** Conceptualization, Data curation, Formal analysis, Funding acquisition, Methodology, Project administration, Resources, Supervision, Validation, Writing – review & editing.

## Funding

Rouen University Hospital.

## Conflict of interests

The authors have no conflict of interests to declare.
