# Supplementary material for: Assessment of the transcriptomic consequences and MAU2 protein levels in edited induced pluripotent stem cells with NIPBL pathogenic variants
Source: Genes Dis. 2024 Aug 6;12(3):101386. doi: 10.1016/j.gendis.2024.101386 (PMC11799743; doi:10.1016/j.gendis.2024.101386)
Supplement: Multimedia component 1 [file mmc1.docx]

**Supplementary methods**

Induced Pluripotent Stem Cells

The iPSC line was previously generated by our group ^1^. This cell line derives from an unaffected male donor. It was analyzed for pluripotency markers expression by quantitative PCR and for chromosomal abnormalities by karyotyping, and was able to differentiate into the three germ layers. Exome sequencing of the parental iPSC line confirmed the absence of rare coding variants in the *NIPBL* and *MAU2* genes. iPSCs were cultured on feeder-free conditions in mTeSR Plus medium (STEMCELL Technologies, Vancouver, Canada) on Matrigel-coated culture dishes (Corning, Corning, NY, USA) diluted in DMEM-F12 according to manufacturer’s instructions in a 37°C/5% CO2 incubator. Cells were split when they reached 80% confluency using StemPro Accutase (Thermo Fisher Scientific) and plated in 10 µM ROCK inhibitor (StemGent, Cambridge, MA, USA) supplemented medium. Medium was refreshed the next day to remove ROCK inhibitor. Cell lines were confirmed to be free of mycoplasma.

Genome editing of iPSCs

*NIPBL* variants were introduced in the genome of iPSC by CRISPR/Cas9. The crispr RNAs (crRNAs) were designed using the CRISPOR.org web tool (http://crispor.tefor.net/). Two crRNAs were designed for each variant. (see additional file). The wild-type Cas9, tracrRNA, crRNA, and ssODN were purchased from IDT (https://eu.idtdna.com). The crRNA and tracrRNA were annealed to form the guide RNA, and then combined to the Cas9 to obtain RNPs. Each RNP and its corresponding single-stranded oligodeoxynucleotide (ssODN) were then nucleofected in the iPSC line, using an AMAXA nucleofector II device. Two days later, cells were diluted and plated into 96-well plates as 0.5 cell/well. When clones reached 80% confluency, genomic DNA was isolated and the presence of the variant was assessed by Sanger sequencing (PCR primers are available upon request). For each variant, 3 clones carrying the variant at the heterozygous state were selected, as well as 3 clones without nucleotide change, corresponding to WT controls. For the R2298C variant, 3 clones carrying the variant at the homozygous state could also be isolated.

RT-ddPCR of NIPBL

Total RNAs were extracted using the Nucleospin® RNA isolation kit (Macherey-Nagel), according to the manufacturer's instructions. RNA was quantified by spectrophotometry (Nanodrop; Thermo scientific). Reverse transcription was performed on 100 ng RNA, using the Verso cDNA kit with oligodT primers (Thermo Scientific). Relative *NIPBL* gene expression in iPSCs was then assessed by digital droplet PCR (ddPCR) on a QX200 plateform (Bio-Rad Laboratories). The ddPCR were performed by relative quantification with TBP, used as reference gene as previously described ^2^. NIPBL was PCR-amplified using the following primers: Fw: 5′-GCCCCATGTCCCCATTAC-3′, Rv: 5′-GCAGGTAAAGGAGATGGAAGAG-3′, associated with the FAM-labeled hydrolysis probe. The reference amplicon, located in the TBP gene, was PCR-amplified using the following primers: (Fw: 5’-CGGCTGTTTAACTTCGCTTC-3’, Rv: 5’-CACACGCCAAGAAACAGTGA-3’) associated with the HEX-labeled hydrolysis probe (IDT DNA). For each cell line, analyses were performed in two technical replicates.

Protein extraction and western blotting

Soluble proteins were extracted from each iPSC clone using RIPA buffer (Pierce, Thermofisher Scientific) and quantified using the DC protein assay kit (Bio-Rad Laboratories). To analyse the NIPBL protein, 30µg proteins were resolved on Tris-acetate NOVEX NuPAGE 3-8% gels (Invitrogen, Thermofisher Scientific). To analyse the MAU2 protein, 20µg proteins were migrated on 10% TGX Stain Free gels (Bio-Rad Laboratories). Proteins were transferred onto a nitrocellulose membrane, blocked in 5% non-fat milk and immunoblotted with the appropriate primary antibody: anti-NIPBL (1:3,000; A301-779A, Bethyl) or anti-MAU2 antibody (1:2,000; Ab183033; Abcam). Membranes were then incubated with secondary peroxidase-labelled anti-rabbit antibody (1:10,000, Jackson Immunoresearch Laboratories). Signals were detected with chemiluminescence reagents (ECL Clarity, Bio-Rad Laboratories) with a GBOX monitored by the Gene Snap software (Syngene). The signal intensity was quantified using the Genetools software (Syngene) and normalized to the total amount of proteins using the Stain-Free signal (ImageLab™ software, Bio-Rad Laboratories).

Patients

Patients selected for the RNAseq analysis from blood samples comprised 8 individuals diagnosed with typical CdLS, carrying heterozygous *de novo* deleterious variants of *NIPBL* identified in their blood by NGS panel sequencing **[Supplementary Table 1]**. The control group for this analysis included 20 subjects, age- and sex-matched with the 8 patients, and without any neurodevelopmental pathologies. Both patients and controls were sampled using PAXgene tubes, and informed consents were obtained from their legal representatives. This study was approved by the Institutional Review Board of the Rouen University Hospital CERDE notification E2023-65).

Transcriptomic data generation and analysis

Total RNAs were isolated from iPSC using the RNeasy kit from Qiagen, or the PAXgene blood RNA kit (Qiagen PreAnalytiX GmbH) for blood samples, according to the manufacturer's recommendations. RNAs were then stored at −80°C until use. The quality and quantity of RNA were assessed using the 4200 TapeStation (Agilent Technologies) and the Qubit 3.0 device (Thermo Scientific). Only RNA samples with a minimal RNA integrity number of 7 were used for subsequent experiments. Libraries were prepared using the NEBNext Ultra II Directional RNA Library Kit for Illumina (New England Biolabs) kit and High-throughput sequencing of the libraries was performed on an Illumina NextSeq 500 (Illumina) using 2*75 bp sequencing to generate 30M read pairs on average per sample. Bioinformatics analysis was carried out using nf-core/RNA-seq v3.1 analysis pipeline to generate multi quality control report that uses the STAR v2.6.1d and SALMON v1.4.0 tools for alignment ^3–5^. Differential analyses were performed using DESeq2 package ^6^ and visual exploration of the BAM files was performed with the IGV tool from the Broad Institute ^7^. For the secondary analysis, the HPO term list was extracted from The Human Phenotype Ontology website (https://hpo.jax.org/app/) ^8^. The list of haploinsufficient genes (pLI > 0.9) was exported from gnomAD v3.1 (https://gnomad.broadinstitute.org) ^9^ and the list of triplosensitive genes (*p*Triplo > 0.9) was obtain from Collins and collaborators ^10^.

**Supplementary figure 1. Variants included in iPSC lines accross the NIPBL gene.**

**
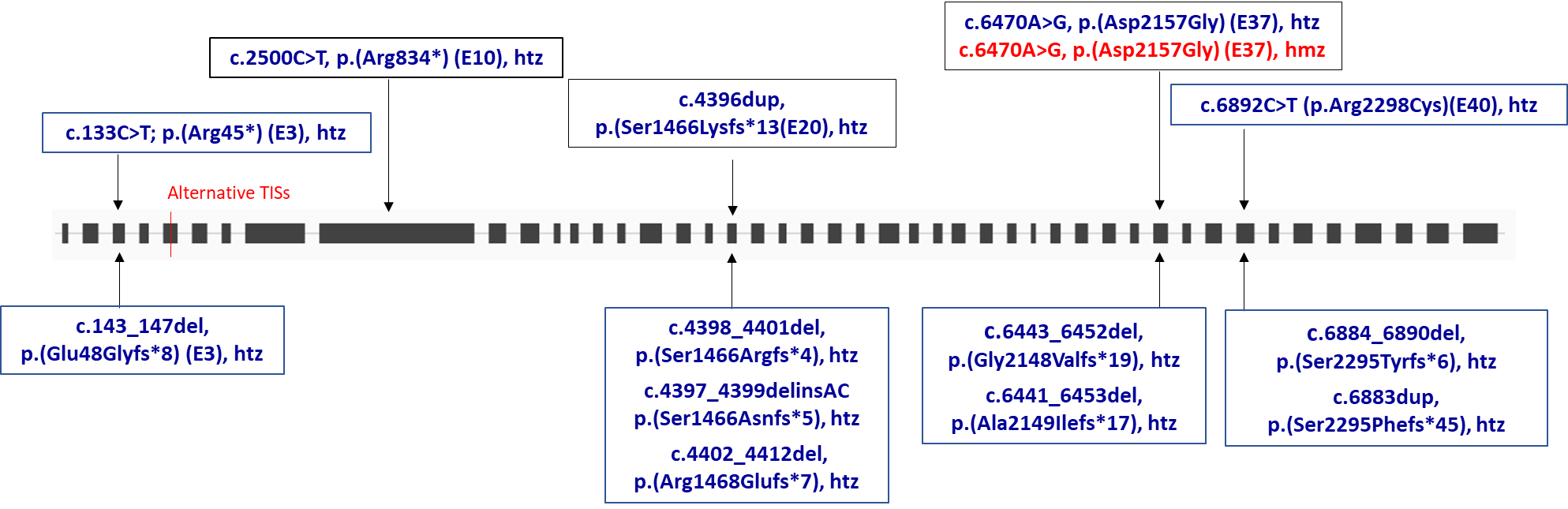
**

Representation of the *NIPBL* gene sequence, where black squares represent the XXX exons and thin lines represent introns. Patient-specific variants are represented with their nomenclature at the cDNA and protein levels at the top of the figure. At the bottom of the figure are represented short insertions or deletions resulting from aberrant DNA repair at the targeted positions. TIS: Translation Initiation Site, htz: heterozygous, hmz: homozygous.

**Supplementary figure 2. Assessment of mRNA levels of NIPBL and MAU2 in the iPSC lines.**


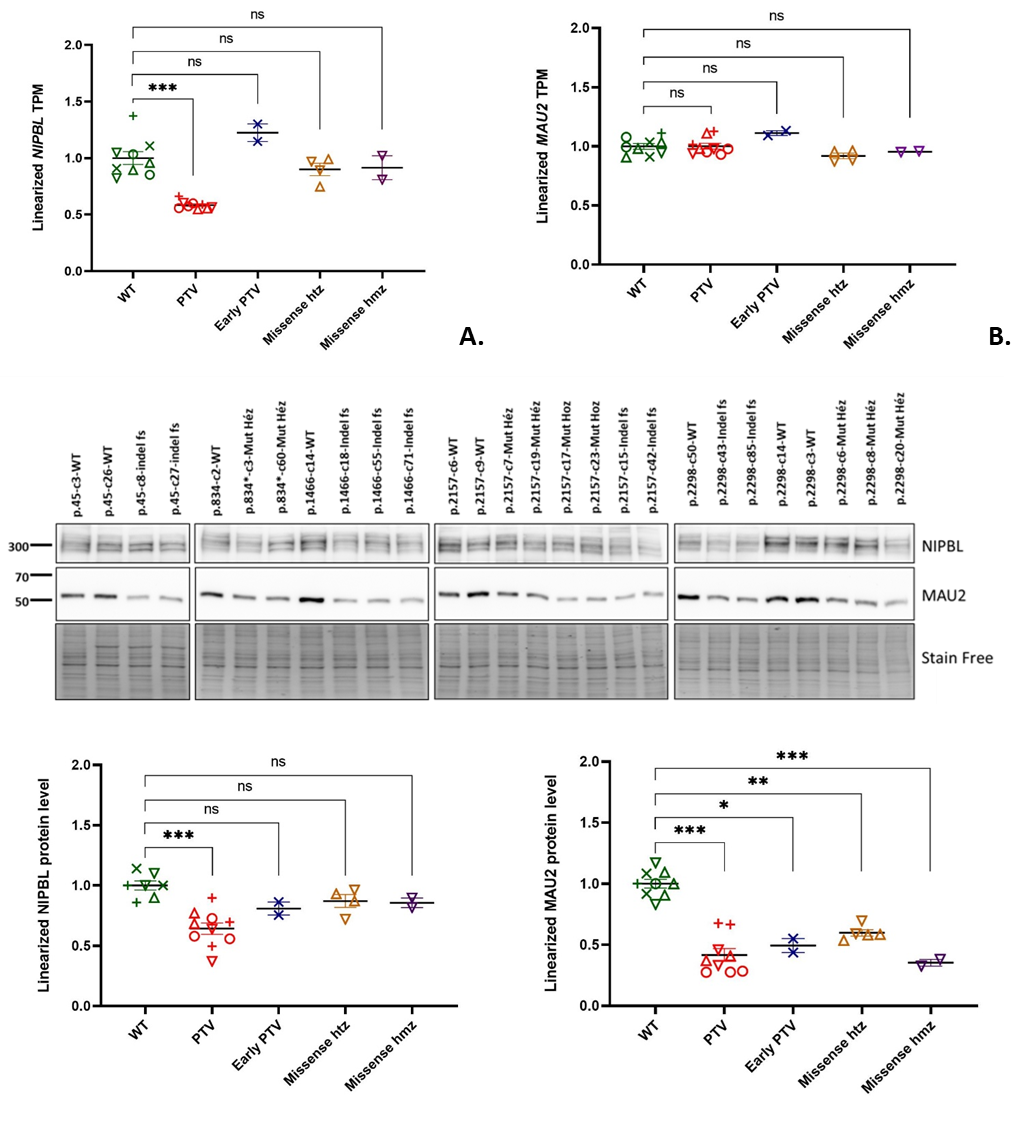


**a, b.** mRNA levels linearized with wild type (WT) iPSC line, expressed in transcript per million (TPM), from RNAseq data for NIPBL (a) and MAU2 (b). For each graph, ✕ represents variants in exon 3 (p.45), + represents variants in exon 10 (p.834), ○ represents variants in exon 20 (p.1466), ▽ represents variants in exon 37 (p.2157) and △ represents variant in exon 40 (p.2298). Green symbols in WT bar represent the WT control of each variant. Horizontal bars represent average and standard deviation. *: *p*<0.05, **: *p*<0.005, ***: *p*<0.001, NS: non-significant.

**Supplementary figure 3. Assessment of protein level of NIPBL in the iPSC lines.**

**
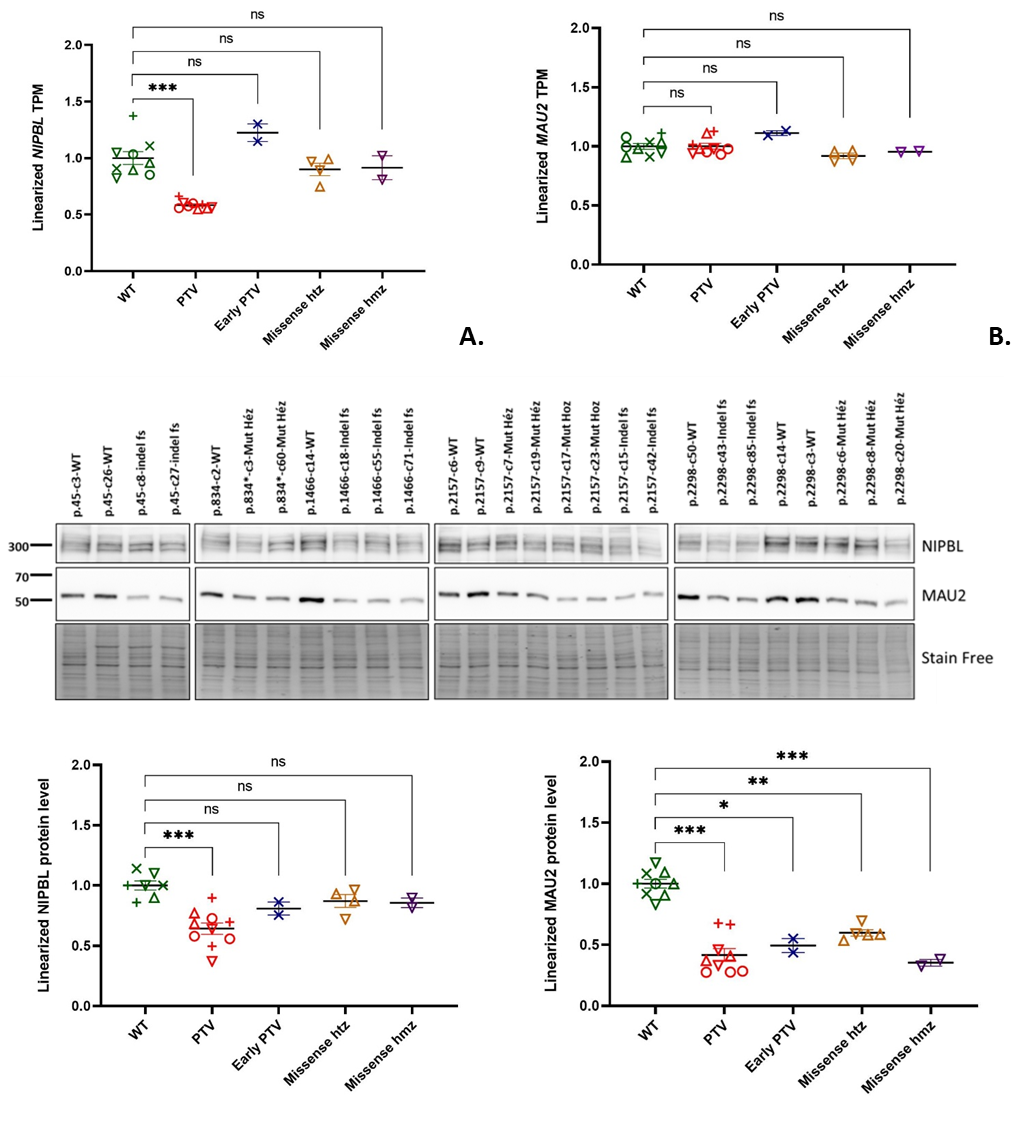
**

Protein levels for NIPBL reported to the stain free signal (figure 1.A). The mean protein levels of the WT lines was arbitrarily set at 1. For each graph, ✕ represents variants in exon 3 (p.45), + represents variants in exon 10 (p.834), ○ represents variants in exon 20 (p.1466), ▽ represents variants in exon 37 (p.2157) and △ represents variant in exon 40 (p.2298). Green symbols in WT bar represent the WT control of each variant. Horizontal bars represent average and standard deviation. *: *p*<0.05, **: *p*<0.005, ***: *p*<0.001, NS: non-significant

**Supplementary figure 4. Results of differential expression analyses, conducted with an FC set at 1.25 and FDR < 0.05.**

HPO overlap between NIPBL and nine dysregulated haploinsufficient OMIM morbid genes, listing all HPO names that NIPBL shares with at least one of the nine genes and highlighting each HPO-gene pair by a colored rectangle.

**Supplemental table 1. List of the nine-downregulated genes, which are both haploinsufficient (pLI > 0.9) and associated with an OMIM Morbid referenced phenotype.**

| **Gène** | **OMIM morbid** | **Syndrome** |
| --- | --- | --- |
| ARFGEF1 | [619964](https://www.omim.org/entry/619964) | Developmental delay, impaired speech, and behavioral abnormalities, with or without seizures; DEDISB |
| CDH11 | [619736](https://www.omim.org/entry/619736) | Teebi hypertelorism syndrome 2; TBHS2 |
| GABRA5 | [618559](https://www.omim.org/entry/618559) | Developmental and epileptic encephalopathy 79; DEE79 |
| GRIA1 | [619927](https://www.omim.org/entry/619927) | Intellectual developmental disorder, autosomal dominant 67; MRD67 |
| GRIK2 | [619580](https://www.omim.org/entry/619580) | Neurodevelopmental disorder with impaired language and ataxia and with or without seizures; nedlas |
| GRIN2A | [245570](https://www.omim.org/entry/245570) | Epilepsy, focal, with speech disorder and with or without impaired intellectual development; FESD |
| NR3C1 | [615962](https://www.omim.org/entry/615962) | Glucocorticoid resistance, generalized; GCCR |
| SATB2 | [612313](https://www.omim.org/entry/612313) | Glass syndrome |
| WNT5A | [180700](https://www.omim.org/entry/180700) | Robinow syndrome |

Among these genes, several are linked to intellectual disability phenotypes and developmental abnormalities, which are central in CdLS. This is the case for *GRIN2A* ^11^*, ARFGEF1* ^12^, *GABRA5* ^13,14^, *SATB2* ^15^ and *WNT5A* ^16^. For most of these genes, seizures are also common. Furthermore, patients carrying pathogenic variants in *GABRA5, SATB2* and *GRIKA* present microcephaly, which is one of the common clinical features of CdLS. Additionally, the *WNT5* gene is associated with Robinow syndrome ^17^, which includes limb abnormalities, as observed in CdLS including clinodactyly of the fifth finger ^18^. On the other hand, *NR3C1* is related to glucocorticoid resistance ^19^, leading to hirsutism in affected individuals. Hirsutism is also present in CdLS, although it does not seem to be related to a hormonal cause; the term hypertrichosis is more commonly used for CdLS. Hypertrichosis is defined as excessive hair growth anywhere on the body in either males or females. It is important to distinguish hypertrichosis from hirsutism, which is a term reserved for females with an excessive amount of terminal hairs in androgen-dependent sites ^20^. Overall, this confluence of signs appears relevant to the CdLS spectrum. However, many of these features are not specific, and the fine mechanisms leading to the above-mentioned syndromes are not purely haploinsufficency for all, so that we cannot claim that CdLS is recapitulated by the intersection of these syndromes.

**Additional reference**

1. Miguel L, Gervais J, Nicolas G, Lecourtois M. SorLA Protective Function Is Restored by Improving SorLA Protein Maturation in a Subset of Alzheimer’s Disease-Associated SORL1 Missense Variants. *J Alzheimers Dis JAD*. Published online July 8, 2023. doi:10.3233/JAD-230211

2. Cassinari K, Rovelet-Lecrux A, Tury S, et al. Haploinsufficiency of the Primary Familial Brain Calcification Gene SLC20A2 Mediated by Disruption of a Regulatory Element. *Mov Disord Off J Mov Disord Soc*. 2020;35(8):1336-1345. doi:10.1002/mds.28090

3. Ewels PA, Peltzer A, Fillinger S, et al. The nf-core framework for community-curated bioinformatics pipelines. *Nat Biotechnol*. 2020;38(3):276-278. doi:10.1038/s41587-020-0439-x

4. Dobin A, Davis CA, Schlesinger F, et al. STAR: ultrafast universal RNA-seq aligner. *Bioinformatics*. 2013;29(1):15-21. doi:10.1093/bioinformatics/bts635

5. Patro R, Duggal G, Love MI, Irizarry RA, Kingsford C. Salmon provides fast and bias-aware quantification of transcript expression. *Nat Methods*. 2017;14(4):417-419. doi:10.1038/nmeth.4197

6. Love MI, Huber W, Anders S. Moderated estimation of fold change and dispersion for RNA-seq data with DESeq2. *Genome Biol*. 2014;15(12):550. doi:10.1186/s13059-014-0550-8

7. Robinson JT, Thorvaldsdóttir H, Winckler W, et al. Integrative Genomics Viewer. *Nat Biotechnol*. 2011;29(1):24-26. doi:10.1038/nbt.1754

8. Köhler S, Gargano M, Matentzoglu N, et al. The Human Phenotype Ontology in 2021. *Nucleic Acids Res*. 2021;49(D1):D1207-D1217. doi:10.1093/nar/gkaa1043

9. Wang Q, Pierce-Hoffman E, Cummings BB, et al. Landscape of multi-nucleotide variants in 125,748 human exomes and 15,708 genomes. *Nat Commun*. 2020;11(1):2539. doi:10.1038/s41467-019-12438-5

10. Collins RL, Glessner JT, Porcu E, et al. A cross-disorder dosage sensitivity map of the human genome. *Cell*. 2022;185(16):3041-3055.e25. doi:10.1016/j.cell.2022.06.036

11. Endele S, Rosenberger G, Geider K, et al. Mutations in GRIN2A and GRIN2B encoding regulatory subunits of NMDA receptors cause variable neurodevelopmental phenotypes. *Nat Genet*. 2010;42(11):1021-1026. doi:10.1038/ng.677

12. Thomas Q, Gautier T, Marafi D, et al. Haploinsufficiency of ARFGEF1 is associated with developmental delay, intellectual disability, and epilepsy with variable expressivity. *Genet Med Off J Am Coll Med Genet*. 2021;23(10):1901-1911. doi:10.1038/s41436-021-01218-6

13. Hernandez CC, XiangWei W, Hu N, et al. Altered inhibitory synapses in de novo GABRA5 and GABRA1 mutations associated with early onset epileptic encephalopathies. *Brain J Neurol*. 2019;142(7):1938-1954. doi:10.1093/brain/awz123

14. Butler KM, Moody OA, Schuler E, et al. De novo variants in GABRA2 and GABRA5 alter receptor function and contribute to early-onset epilepsy. *Brain J Neurol*. 2018;141(8):2392-2405. doi:10.1093/brain/awy171

15. Leoyklang P, Suphapeetiporn K, Siriwan P, et al. Heterozygous nonsense mutation SATB2 associated with cleft palate, osteoporosis, and cognitive defects. *Hum Mutat*. 2007;28(7):732-738. doi:10.1002/humu.20515

16. Roifman M, Marcelis CLM, Paton T, et al. De novo WNT5A-associated autosomal dominant Robinow syndrome suggests specificity of genotype and phenotype. *Clin Genet*. 2015;87(1):34-41. doi:10.1111/cge.12401

17. Patton MA, Afzal AR. Robinow syndrome. *J Med Genet*. 2002;39(5):305-310. doi:10.1136/jmg.39.5.305

18. Abu-Ghname A, Trost J, Davis MJ, et al. Extremity anomalies associated with Robinow syndrome. *Am J Med Genet A*. 2021;185(12):3584-3592. doi:10.1002/ajmg.a.61884

19. Bray PJ, Cotton RGH. Variations of the human glucocorticoid receptor gene (NR3C1): pathological and in vitro mutations and polymorphisms. *Hum Mutat*. 2003;21(6):557-568. doi:10.1002/humu.10213

20. Saleh D, Yarrarapu SNS, Cook C. Hypertrichosis. In: *StatPearls*. StatPearls Publishing; 2023. Accessed August 20, 2023. http://www.ncbi.nlm.nih.gov/books/NBK534854/
